# Perineal pruritus in epidural dexamethasone injections

**DOI:** 10.1080/24740527.2019.1650614

**Published:** 2019-08-28

**Authors:** Catherine Veilleux, Aline Boulanger

**Affiliations:** Faculté de médecine et des sciences de la santé, Université de Sherbrooke, Sherbrooke, Québec, Canada; Département d’anesthésiologie, Faculté de médecine, Université de Montréal, Montréal, Québec, Canada

Intravenous dexamethasone has been reported to induce short-lived sensations such as burning, itching, and tingling in the perineal region,^1–4^ with most cases reported among women. The mechanism behind this reaction is currently unknown and not all types of corticosteroids have been associated with this adverse reaction.^5^ It has been postulated that this adverse reaction may be caused by the phosphate ester of the dexamethasone sodium phosphate,^4^ providing an explanation for why it has not been reported with intravenous methylprednisolone injections. There are only a few reports of burning sensations in the perineal region with epidural dexamethasone injections. One case of generalized pruritus originating from the groin area has been reported after a transforaminal epidural dexamethasone injection.^6^ Much like adverse reactions described with intravenous injections, the reaction described began soon after the injection was completed and was short-lived. The authors of this case report then conducted a prospective study to determine the adverse effects immediately following transforaminal epidural steroid injection for the treatment of radicular pain.^7^ Their results showed that 7 out of 150 patients (4.6% of patients) experienced a similar short-lived perineal pruritus following procedures involving epidural dexamethasone injection. This adverse effect was not reported with epidural injections with steroids other than dexamethasone. The authors proposed that this adverse effect was possibly related to a rapid injection of the medication, an unrecognized vascular injection, or both mechanisms.^7^

We herein present the case of a 60-year-old woman who received interlaminar epidural injections every 4 months between 2006 and 2016 to alleviate lumbar radicular pain caused by degenerative disc disease at the level of L4–L5 and by disk herniation at the level of L5–S1. She had no previous history of allergy. These epidural injections performed on a regular basis decreased her pain and allowed her to remain functional at a full-time job. Between 2006 and 2010, this patient received analgesic epidural injections of methylprednisolone 80 mg (methylprednisolone acetate injectable suspension USP, Pfizer, 80 mg/mL, 1 mL per vial) diluted in 1 mL of lidocaine 2% (lidocaine 2%, Aspen Pharmacare Canada), and 4 mL of normal saline (normal saline 0.9% injectable USP, Hospira). In January 2011, following emerging case reports of paraplegia provoked by suspension particles in methylprednisolone preparations,^8,9^ the epidural preparation was changed to dexamethasone 10 mg (dexamethasone sodium phosphate, Omega, 10 mg/cc, 1 cc/vial) diluted in 1 mL of lidocaine 2% and 4 mL of normal saline. All three products were preservative free. Between January 2011 and April 2015, the patient received a total of 14 injections containing dexamethasone. On April 15, 2015, soon after the epidural injection, the patient reported experiencing transient vulvar burning sensations. Upon further enquiry, the patient admitted to having experienced similar symptoms with each of her injections between January 2011 and April 2015. She described the adverse reaction as a burning sensation around the vulvar region, which began seconds to minutes after the injection and lasted no more than a few minutes. This burning sensation then disappeared and was not accompanied by any other neurological symptoms. The patient also revealed that she was worried that the pain could persist or be associated with more severe neurological complications over time. At that point, the epidural injections were changed back to methylprednisolone 80 mg in 1 mL 2% lidocaine and 4 mL of normal saline. Following this change in her epidural injections, the patient did not experience this unpleasant adverse effect again.

A limitation in determining the origin of the symptoms associated with the injections was that they were not conducted under fluoroscopic guidance. We can therefore not rule out vascular injection, but this would be unlikely given the history that the same adverse effect reoccurred after every injection. We suspect the burning sensation to be a result of dexamethasone-induced idiosyncratic adverse reaction.

We believe that perineal pruritus following epidural injections with dexamethasone may be underreported due to patients’ fear of embarrassment in reporting such an adverse effect. Our case suggests that this finding appears to be a local adverse effect from dexamethasone injection rather than from intravascular penetration because it was reported to occur after every injection for several years. Our observation further suggests that physicians should be aware of this adverse effect, which can be stressful and debilitating. Both observational studies and clinical trials using dexamethasone for epidural injections should consider eliciting and reporting this adverse event in their population.
